# Correlation between Long-Term Aspirin Use and F-Fluorodeoxyglucose Uptake in Colorectal Cancer Measured by PET/CT

**DOI:** 10.1371/journal.pone.0109459

**Published:** 2014-10-07

**Authors:** Binbin Su, Baixuan Xu, Jun Wan

**Affiliations:** 1 Department of Gastroenterology, South Building, Chinese PLA General Hospital, Beijing, China; 2 Department of Nuclear Medicine, Chinese PLA General Hospital, Beijing, China; University General Hospital of Heraklion and Laboratory of Tumor Cell Biology, School of Medicine, University of Crete, Greece

## Abstract

**Purpose:**

The aim of this study was to evaluate the relationship between long-term aspirin use with pretreatment 18 Fluorodeoxyglucose (FDG) uptake of primary lesions of Colorectal cancer (CRC) and evaluate their clinical significance.

**Materials and Methods:**

We enrolled 84 patients with CRC who underwent 18F-FDG PET/CT scanning before surgery between 1st July 2008 and 1st March 2013 and followed up until 1st March 2014. Maximum standardized uptake value (SUVmax) of the primary tumor was measured by 18F-FDG PET/CT. The history of aspirin taken and other clinicopathogical factors were also obtained and their relationships were examined by Mann-Whitney or χ2 tests. Progression-free survival (PFS) was determined by standard Kaplan-Meier survival analysis. Cox proportional hazards regression was performed to determine whether history of aspirin taken, pretreatment SUVmax, age, gender, TNM stage, tumor sizes and differentiation influenced outcomes.

**Results:**

CRC Patients with long-term history of aspirin use had lower SUVmax of primary lesions than control group (9.74±2.62 vs. 13.91±6.18) and showed a trend towards improved PFS after curative surgery. However, pretreatment of SUVmax showed no prognostic value in patients with CRC.

**Conclusions:**

Long-term aspirin use is associated with lower pretreatment SUVmax of CRC and is a promising prognostic factor for predicting PFS in patients with CRC.

## Introduction

Colorectal cancer (CRC) is the third most common cancer in the world, accounting for more than 1.2 million new cases diagnosed and 600,000 deaths annually [1]. There is a substantial body of epidemiological evidence indicating that regular use of aspirin is associated with a reduced risk of CRC-specific mortality [2]. More recently, a meta-analysis of five large cardiovascular trials provides proof-of-principle that aspirin is able to reduce distant cancer metastasis [3]. However, the mechanism of action of the CRC chemopreventive and anticancer effects of aspirin is not fully understood and debatable [4].

Biological imaging by positron emission tomography (PET) using 18F-2-fluoro-2-deoxy-D-glucose(FDG) has been widely used clinically for the detection of primary tumors and for early prediction of response to chemotherapy [5]. Given the known biological characters of tumors, 18F-FDG accumulation in tumors is used as index of increased glucose uptake and as a marker of tumor proliferation and metastatic potential, and recent investigations demonstrate the usefulness as a prognosticator for eventual outcomes [6], [7], [8].

Consistent with experimental evidence in animals that aspirin can impair glucose transport in tumor cells by targeting the pivotal glucose transporter GLUT1 [9], which is directly involved in FDG uptake, we therefore hypothesized that long term aspirin use may mediate changes of CRC biological characteristic which could be evaluated by FDG uptake. Based on that, this study was initiated to evaluate FDG uptake of CRC patients with long-term history of aspirin use and determined whether metabolic activity is correlated with recurrence and progression-free survival.

## Materials and Methods

### Patients

This study was approved by the Institutional Review Board of Chinese PLA General Hospital, and the requirement of informed consent was waived due to the retrospective nature of this study. From 1st July 2008 and 1st March 2013, patients that presented to our institution with the initial diagnosis of CRC and were submitted to a curative CRC resection were retrospectively reviewed. Patients recruitment criteria included: 1) patients had a baseline PET/CT scan within 2 weeks prior to surgery, 2) they had radical surgery with stage I to III, 3) they did not received any neoadjuvant chemotherapy or radiation therapy before undergoing 18F-FDGPET or surgery. The pathological stage of the disease was based on the pathology reports from the definitive surgery in all cases. From the medical records, the history of aspirin use was documented. The patients were divided into two groups. The patients who have regular aspirin taken for at least 5 years with doses of at least 75 mg daily comprised the aspirin group, while the patients who never or occasionally take aspirin were comprised the control group.

### PET/CT acquisition and analysis

All subjects underwent PET/CT scans using 18F-FDG within 2 weeks before surgery with the protocol of PET/CT scanning used at our institution. Before imaging, patients fasted for 6 h and their blood glucose levels were as tested, below 7 mmol/L. The recommended intravenously injected dosage for 18F-FDG was 5.55 MBq/kg and a standard 60 min post-injection rest allowed before PET/CT scanning. FDG-PET/CT scans were performed on a PET/CT scanner (Biograph Truepoint, Siemens). The data acquisition procedures were as follows: The scan covered the trunk from skull base to midthigh. Attenuation correction was performed using a low-dose helical CT protocol (90 mAs, 110 kV, 0.9 pitch) under normal breathing. Immediately after CT scanning, a PET emission scan that covered the identical transverse field of view was obtained. The PET images, including axial, sagittal and coronal images, were reconstructed using the true X method, and postfiltered with a 5.0 mm full width at half maximum (FWHM) in a matrix of 168. SUV = (activity/unit volume)/(injected dose/total body weight). The maximum SUV (SUV max) was defined as the peak SUV on one pixel with the highest counts within region of interest. Images of PET/CT were reviewed by two nuclear medicine doctors experienced in PET/CT image reading.

### Histological analysis and outcome evaluation

All surgical specimens were diagnosed by one experienced pathologist, and the longest diameter reported by the pathologist was used as the tumor size in this analysis. Tumor-related parameters were collected including p-T stage, p-N status, tumor size and histopathological findings.

After curative resection of the tumor, adjuvant chemotherapy or chemoradiotherapy were indicated for all patients with lymph node-positive colorectal cancer. All patients had follow-up examinations approximately every 3 months for the first 1 year, every 6 months for the next 2 years, and every 1 year thereafter. Tumor recurrence was confirmed by either tissue biopsy or the demonstration of progressive disease by serial imaging methods. Clinical proofs of no recurrent disease consisted of a negative physical and examination, negative tissue biopsy, and negative findings on serial follow-up imaging methods.

### Statistical analysis

Group means were compared using the Mann-Whitney or χ2 tests. Progression-free survival was defined as the time to a specified recurrence and was measured from the day of surgery. Progression-free survival (PFS) was determined by standard Kaplan-Meier survival analysis, and between-group comparison was performed by log-rank test. Hazard ratios (HR) were derived from Cox regression analysis. All statistics were completed using SPSS version 20 (IBM Corp, Armonk, NY). A P-value less than 0.05 was considered statistically significant.

## Results

### Patient characteristics

There were a total of 1023 cases with CRC who were diagnosed and treated in the authors’ institution between 1st July 2008 and 1st March 2013. Eighty-four patients with a median age 72 years (range: 32–89 years) were identified. The baseline characteristics of the patients are shown in Table 1. Sixty two (73.8%) patients were male. Aspirin group comprised 23.8% of patients. All of those 20 patients took aspirin at dose of 100 mg per day for CV disease prevention. The pretreatment maximum SUV ranged from 4.9 to 32.0 with median value of 13.0. There were no statistically significant differences between the aspirin and control groups with respect to any of clinicopathological factors.

**Table 1:** Patient demographics and clinical characteristics.

Characteristic	Aspirin group n = 20	Control group n = 64	P-value
Age (y)	75.2±9.87	71.7±13.8	0.30
Gender			
Male	16(80%)	46(71.9%)	0.47
Female	4(20%)	18(28.1%)	
Tumor sizes (cm)	4.26±1.80	4.87±2.16	0.25
Differentiation			
Well	15(75%)	51(79.7%)	0.65
Poor	5(25%)	13(20.3%)	
T classification			
T1+T2	4(20%)	13(20.3%)	0.97
T3+T4	16(80%)	51(79.7%)	
N classification			
N0	13(65%)	43(67.2%)	0.85
N1+N2	7(35%)	21(32.8%)	
Pretreament SUV max	9.74±2.62	13.91±6.18	0.004
Dose of aspirin (mg/d)	100	0	0

### The association between Long term aspirin use and SUV max

Detailed results for the relationships between 18F-FDG uptake and clinicopathological factors are shown in Table 2. Long term aspirin use was found to have a significant relationship with 18F-FDG uptake (p = 0.04). Lower 18F-FDG uptake was observed in aspirin group compared with control group, with statistically significant differences in SUVmax. In addition, High p-T stage (p = 0.02) and large tumor sizes (p = 0.02) were associated with increases in SUVmax. Finally, there were no statistically significant differences in SUV max in terms of other histopathological findings. Gender, age (<60 years versus ≥60 years), p-N status, and differentiation were not associated with the SUV max.

**Table 2:** Clinicopathological implication of Pretreament SUVmax combination status.

Characteristic	No. of patients	Pretreament SUVmax	P-value
Age (y)			
<60	14	14.0±5.56	0.4
≥60	70	12.6±5.87	
Gender			
Male	62	13.2±6.45	0.4
Female	22	12.0±3.35	
Tumor sizes (cm)			
<5	49	11.2±5.03	0.02
≥5	35	15.2±6.12	
Differentiation			
Well	66	12.78±5.73	0.67
Poor	18	13.4±6.24	
T classification			
T1+T2	17	10.1±4.17	0.02
T3+T4	67	13.63±5.97	
N classification			
N0	56	13.5±6.26	0.17
N1+N2	28	11.7±4.65	
Aspirin use			
Yes	20	9.74±2.62	0.04
No	64	13.91±6.18	

### Long term aspirin use as a predictor of survival

Based on the SUV max cutoff value of 13.0 (4.9–32.0), patients were divided into a high SUV max group (SUV max ≥13.0; n = 37) or a low SUV group (SUV max<13.0, n = 47). The median follow-up was 52.7 months (range 0.7–70.3). During this period, 21 patients showed recurrent disease, of which 13 patients had distant metastases, 4 patients had both local and distant recurrence, 4 patients died from cancer-related causes.

**Figure 1:**
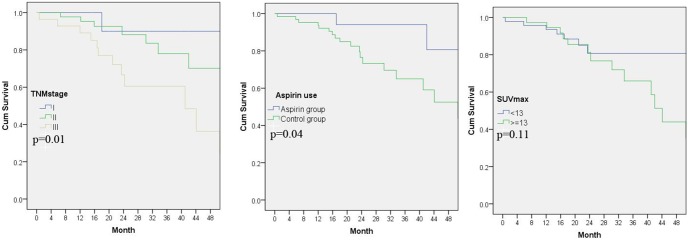
Progression-free survival as differentiated by TNM stage, history of aspirin use and SUVmax, respectively. A: PFS differences between TNM categories, p = 0.01. B: PFS differences between aspirin categories, p = 0.04; C: PFS differences between SUV max categories, p = 0.11.

Aspirin group had a statistically significant improvement in PFS compared to control group. The corresponding 2-year probability of PFS was 94% and 73%, respectively (Fig. 1). In contrast, SUV max showed no significant association with PFS (p = 0.11).

In the univariate analysis (Table 3), N stage and tumor differentiation showed a significant associations with PFS (p = 0.005, P = 0.002, respectively). However, long term history of aspirin use also showed a trend towards improved PFS (P = 0.06). At multivariate analysis, including N stage, aspirin use history and tumor differentiation, only N stage showed a prognostic significance (p = 0.04).

**Table 3:** Univariate and multivariate analysis of prognostic factors of PFS.

Characteristic	Univariate analysis			Multivariate analysis		
	HR	95%CI	P	HR	95%CI	P
Age (<60 vs ≥60)	0.45	0.21–1.97	0.453			
Gender (M vs F)	0.65	0.23–1.81	0.414			
Sizes (<5 cm vs ≥5 cm)	0.871	0.38–2.25	0.929			
Dif (well vs poor)	4.121	1.666–10.194	0.002	2.47	0.914–6.689	0.075
T (T1+2 vs T3+4)	1.624	0.476–5.548	0.439			
N (N0 vs N1+N2)	3.578	1.45–8.77	0.005	2.696	1.008–7.209	0.048
Aspirin (yes vs no)	4.10	0.945–17.82	0.06	3.58	0.81–5.884	0.093
SUVmax (<13 vs ≥13)	2.061	0.822–5.17	2.06			

## Discussion

Epidemiological studies have provided information on the chemopreventive effect of aspirin against CRC [10]. However, the optimal dose and duration of aspirin is not known. On the basis of current evidence, it can be assumed that regular dose of aspirin (75 mg-300 mg) taken over a 5-year period in patients will probably confer substantial CRC-specific survival benefit [11]. Thus, in this study, we identified aspirin group as the patients who have at least 5-year history of aspirin use at dose of 75 mg–300 mg daily.

To our knowledge, no research has been conducted on the relationship between aspirin use and FDG uptake in CRC. However, the interest in FDG-PET to assess the response of tumor to chemotherapy began in the early 1990s [12]. Previous studies have already shown that the degree of chemotherapy-induced metabolic changes in tumors is highly predictive of patient outcome [13], [14]. In the past 5 years, multiple lines of evidence indicate that regular aspirin use can reduce CRC-specific mortality [15], [16], [17]. However, aspirin show no demonstrable activity in patients with overt metastatic cancer [18], [19]. Furthermore, in a recent large population-based cohort, low-dose aspirin usage after diagnosis of CRC did not increase survival time [20]. The differential effects of aspirin before and after diagnosis of CRC probably indicated that long term use of aspirin before diagnosis may mediate tumor biological changes to less aggressive type, which may be evaluated by the SUV in FDG-PET. In an attempt to throw some light on this, we conducted our survey.

In the present study, SUV max was significantly related to tumor size and depth of invasion, which is in agreement with most clinical studies on various malignancies, such as colon cancer [21], pancreas cancer [22] and breast cancer [23]. More important, according to our data, long term aspirin use before diagnosis of CRC is negatively correlated with FDG uptake of primary lesions and showed significant improvement in PFS.

As known, FDG uptake is used as index of increased glucose metabolism and as a marker of tumor viability for which, the degree of FDG uptake presumed to reflect tumor aggressiveness [24], [25], [26]. Several studies have demonstrated that FDG uptake is an indirect reflection of tumor hypoxia [27]. Hypoxia renders tumor cells more invasive phenotype and less susceptible to radiotherapy and chemotherapeutic agents [28], [29]. This is substantiated by clinical studies which revealed that tumor hypoxia is associated with poor disease-free and overall survival rates in several types of cancer [30]. FDG also reflect tumor differentiation [31] and genetic mutation status [32]. In a study focusing on CRC, SUV on pre-treatment F-18 FDG-PET/CT could be used as a good surrogate marker for the prediction of disease progression [33]. Therefore, FDG uptake is now considered to be a useful surrogate in-vivo biomarker in the staging, predicting tumor aggressiveness and treatment outcome.

Although direct effect of aspirin on FDG uptake is still an unexplored preposition, emerging evidence indicated that aspirin is involved in shaping tumor microenvironment which may have an impact on FDG uptake [34]. As known, aspirin inhibits both COX-1 and COX-2; but it preferentially inhibits COX-1. Because platelets have a limited capacity to generate COX-1 de novo [35], the oral administration of aspirin at low-dose, once daily causes an almost complete suppression of platelet COX-1 [36]. On the contrary, very high doses of aspirin (>1000 mg), do achieve sufficient systemic concentrations to inhibit COX-2 activity [37], and this inhibition can only be maintained by repeated dosing three or four times daily [38]. In addition, higher dose of aspirin do not further improve CRC survival [39]. The finding of aspirin benefit at low dose in cancer prevention, locates the antiplatelet effect of aspirin at the center of its antitumor efficacy. To date, at least 20 angiogenic-regulating factors have been identified in platelets, such as platelet-derived growth factor (PDGF), vascular endothelial growth factor (VEGF), fibroblast growth factor (FGF), angiostatin and insulin-like growth factor (IGF), including both pro-and anti-angiogenic factors [40]. Excess production of pro-angiogenic factors and/or diminished production of anti-angiogenic molecules were considered responsible of tumor vascular abnormality and hostility of tumor microenvironment [41], [42]. Restoration of pro-and anti-angiogenic balance in tumors may “normalize” tumor vasculature and thus indirect reduce the hostility of TME [43]. Given an abundant array of positive and negative angiogenesis regulators contained in platelets, platelets may have capacity to stimulate every stage of tumor angiogenesis and to shape TME. In fact, a recently study directly demonstrated that aspirin alter tumor microenvironment in T-cell lymphoma mouse model, which showed that oral administration of aspirin to mice as a prophylactic measure was accompanied by alterations in the biophysical, biochemical and immunological composition of the tumor microenvironment with respect to PH, level of dissolved O2 and glucose [44].

In present study, we found aspirin’s ability to decrease the SUV max of primary CRC lesions, while demonstrated no prognostic information from SUVmax of the primary lesions. Despite some researchers have suggested that FDG-PET have prognostic value for some types of malignancies [45], [46], [47], past studies were mainly focused on stage IV CRC [48]. Our study included only resectable CRC and is consistent with results from Lee.JE [49]. Use of aspirin prior to diagnosis was found to be associated with earlier tumor stage for CRC [50], [51], however, it was beyond the scope of this study to assess whether long term use of aspirin provides a favorable impact on the staging characteristics of CRC. The main prognostic factors in this study for PFS were tumor stage, especially nodal stage.

There are some limitations in this study: first, the size of this study was relatively small. This was due to the fact that PET/CT scanning before CRC surgery is not the protocol in our institution; second, the duration of follow-up was relatively short. However, a recent analysis showed that most relapses occur within 2 years of surgery. Third, immunohistochemical information on glucose metabolism in colorectal cancer was unavailable. Therefore, a biological explanation for the correlation between aspirin and FDG accumulation and the behavior of the tumor was not suggested.

## Conclusion

In conclusion, this study is a relatively small analysis, but highlights the fact that FDG accumulation in primary CRC with long term aspirin use is significantly lower than in those without aspirin use. Although the larger number of patients is needed, these results indicate that aspirin may mediate changes of CRC biological characteristic. Thus, this study provides a novel possibility of FDG-PET/CT scans to be used for noninvasive evaluation for cancer prevention strategies. To validate this idea, further prospective studies with a larger number of patients and clinical follow-up are needed. Furthermore, it should be investigated whether FDG-PET/CT scans might predict the actual response to aspirin as well as survival rates.
